# Prevalence and sociodemographic disparities in ever E-cigarette use among adults in Los Angeles County

**DOI:** 10.1016/j.pmedr.2019.100904

**Published:** 2019-05-22

**Authors:** Yajun Du, Margaret Shih, Megha D. Shah, Mark D. Weber, Amy S. Lightstone

**Affiliations:** a Los Angeles County Department of Public Health, Office of Health Assessment & Epidemiology, Los Angeles, CA 90012, United States of America; b UnitedHealthcare, Minnetonka, MN, United States of America; c Los Angeles County Department of Public Health, Tobacco Control and Prevention Program, Los Angeles, CA 90010, United States of America

**Keywords:** Electronic cigarette, Prevalence, Adults, Los Angeles County

## Abstract

E-cigarette use has increased rapidly among U.S. adults. Few studies have examined the prevalence and risk factors of e-cigarette use among adults in the United States. We conducted descriptive analyses to identify characteristics of ever e-cigarette users among adults (18 years and older) living in Los Angeles (LA) County, the most populous county in the U.S., using data from the 2015 LA County Health Survey. We used logistic regression to examine independent factors associated with ever e-cigarette use. Age-adjusted prevalence of ever e-cigarette use was 8.4%. A significant interaction between conventional cigarette smoking status and gender was found (p < 0.05), and several factors were identified as associated with ever e-cigarette use in models stratified by gender. Various social and demographic factors are associated with ever e-cigarette use and should be incorporated into evidence-based interventions.

## Highlights

• In LA County, 11.6% of males and 5.7% of females reported ever using e-cigarettes.
• The prevalence of ever e-cigarette use was 18.5% among young adults (18–24 years).
• Factors associated with ever e-cigarette use varied by gender.
• Concurrent use of other substances was associated with ever e-cigarette use.

## 1 Background/objective

Conventional cigarette smoking in the U.S. has fallen to an all-time low since 1964, when the first Surgeon General's report was issued (CDC, 2014; Norris et al., 2018; Wang et al., 2018), and has also fallen significantly in LA County (OHAE, 2010), contrasting with emerging electronic cigarette (e-cigarette) use in the US (CDC, 2014; Agaku et al., 2014). In 2014, 12.8% of U.S. adults reported ever e-cigarette use (Wilson and Wang, 2017). The comparative harms and benefits of using e-cigarettes have been widely debated by public health professionals (CDC, 2016). Although no overall consensus has been reached, the rapid increase in e-cigarette use among youth and young adults is considered a major public health concern (PHE, 2015). However, little is known about the sociodemographic characteristics of e-cigarette use among adults. LA County is one of the few local health jurisdictions with a local population-based health survey. This diverse metropolitan region, which has the largest county population in the U.S., comprises a broad range of sociodemographic subpopulations, and allows the examination of health problems and health disparities among unique population subgroups. It also provides local public health officials, community organizations, and policymakers with critical data that can be used to inform local public health programs and policy decisions. The primary objective of our study was to investigate the prevalence of ever e-cigarette use among adults in LA County; the secondary objective was to examine sociodemographic factors associated with ever e-cigarette use among adults, overall and by gender.

## 2 Methods

Data are from the most recent cycle of the LA County Health Survey (2015), a population-based random digit dial telephone survey (landline and cell phone). Interviews were conducted in English, Spanish, Chinese (Mandarin and Cantonese), Korean, and Vietnamese. Statistical weighting is utilized to generalize the sample survey data to the overall LA County population (Abt and SRBI, 2017).

Outcome measures: ever e-cigarette use was measured by the question, “Have you ever smoked electronic cigarettes? (yes/no)”.

Independent variables of interest included gender (female/male), age (18–24 years; 25–49 years; 50 years and older), race/ethnicity (white, Latino, black, Asian), disability (no/yes), nativity (US born/foreign born), education (less than high school; high school graduate; some college or higher), the federal poverty level (FPL) (0–99% FPL; 100–299% FPL; ≥300% FPL), housing instability (i.e., reported being homeless or not having their own place to live or sleep) in the past 5 years (no/yes), marijuana use in the past year (no/yes), alcohol consumption in the past month (non-drinkers; non-heavy or non-binge drinkers; heavy or binge drinkers) (CPSTF, 2017), and cigarette smoking status (never smoked/non-regular smoker; former smoker; current smoker).

We conducted descriptive analyses to identify demographic characteristics of ever e-cigarette users. Age-adjusted prevalence of ever e-cigarette use was calculated using the 2000 US standard population. Seven age groups were used for age adjustment among adults: 18–24, 25–29, 30–39, 40–49, 50–59, 60–64, and 65 or over. We performed multivariable logistic regression to examine the associations between ever e-cigarette use and demographic characteristics (age, gender, race/ethnicity), socio-economic status (education, FPL, housing instability), disability status, nativity (US born vs. foreign born), and the use of other substances (alcohol, marijuana, cigarettes). An interaction between two independent variables (cigarette smoking status and gender) was significantly associated with ever e-cigarette use (p < 0.05), consequently, separate logistic models were developed for females and males. A significance level of α = 0.05 was used for statistical testing. All analyses were conducted using Statistical Analysis System (SAS), version 9.3 (SAS Institute Inc., Cary, NC).

## 3 Results

### 3.1 Characteristics of study sample (unweighted)

A total of 8008 adults (ages 18 years or older) residing in LA County were interviewed (5026 landlines and 2982 cell phones) in 2015. The survey cooperation rate was 69.0% (Table 1). Native Hawaiian and other Pacific Islanders and American Indian/Alaska Native (n = 89) were excluded from our study due to small sample size. Females represented 59.6% of the sample, and the median age was 54 years. Age groups: 7.7% were 18–24 years old, 33.2% were 25–49 years old, and 59.1% were 50 years or older. Approximately one-third (33.5%) were Latino, 44.3% were white, 12.6% were black, 9.6% were Asian.

**Table 1:** Demographic characteristics of the study population (weighted) among LA County adults^COOP^, overall and by gender.

Characteristic	Overall (n = 7919)	Male (n = 3198)	Female (n = 4721)
	Weighted % (95% CI)	Weighted % (95% CI)	Weighted % (95% CI)
LA County	100.0	48.8 (47.3, 50.4)	51.2 (49.6, 52.7)
Age (years)			
18–24	14.1 (12.8, 15.3)	14.7 (12.9, 16.5)	13.5 (11.9, 15.1)
25–49	46.8 (45.3, 48.4)	48.2 (45.9, 50.6)	45.5 (43.4, 47.6)
50+	39.1 (37.7, 40.5)	37.1 (35.0, 39.2)	41.0 (39.1, 42.9)
Race/ethnicity^R^			
White	31.3 (30.0, 32.7)	32.4 (30.4, 34.4)	30.3 (28.6, 32.0)
Latino	44.3 (42.8, 45.9)	42.5 (40.1, 44.8)	46.1 (44.0, 48.1)
Black	8.8 (8.0, 9.5)	8.0 (6.9, 9.1)	9.5 (8.5, 10.5)
Asian	15.6 (14.3, 16.8)	17.1 (15.2, 19.1)	14.1 (12.4, 15.7)
Education			
Less than high school	22.5 (21.1, 24.0)	20.3 (18.2, 22.4)	24.6 (22.7, 26.6)
High school	21.5 (20.2, 22.8)	23.2 (21.1, 25.2)	19.9 (18.2, 21.6)
Some college or higher	56.0 (54.4, 57.6)	56.5 (54.1, 58.9)	55.5 (53.4, 57.5)
Household income^FPL^			
0–99% FPL	22.5 (21.1, 23.8)	18.1 (16.3, 20.0)	26.6 (24.7, 28.5)
100–299% FPL	39.1 (37.6, 40.7)	39.5 (37.2, 41.8)	38.8 (36.8, 40.8)
300% or above FPL	38.4 (37.0, 39.9)	42.4 (40.1, 44.6)	34.6 (32.8, 36.5)

^COOP^Cooperation rate was 69.0%, which was the number of complete interviews (I) divided by the number of interviews (complete plus partial: I + P) plus the number of refusal (R). Cooperation Rate = I / ((I + P) + R)).

^R^Due to small numbers, Native Hawaiian and Other Pacific Islanders (NHOPI) and American Indian/Alaska Native (AI/AN) were excluded.

^FPL^Based on U.S. Census 2013 Federal Poverty Level (FPL) thresholds.

### 3.2 Characteristics of study population (weighted), overall and by gender

We compared our survey sample (weighted) demographics with LA County Population Estimates by survey year and found the weighted sample closely reflected the demographic makeup of LA County's adult population (Du et al., 2017). Detailed characteristics of the study population (weighted), overall and by gender are shown in Table 1.

### 3.3 Age-adjusted prevalence of ever e-cigarette use among LA County adults, overall, by gender, and by race/ethnicity

Age-adjusted prevalence of ever e-cigarette use was 8.4% among LA County adults. Prevalence of ever e-cigarette use among males was higher than among females (11.1% vs. 5.7%, respectively). Prevalence of ever e-cigarette use was the highest among whites (12.8%), followed by Asians (8.9%), Latinos (6.1%), and blacks (5.8%) (Table 2).

**Table 2:** Age-adjusted^a^ prevalence of ever e-cigarette use among LA County adults, overall, by gender, and by race/ethnicity, education and household income.

Characteristic	Overall	Male	Female
	% (95% CI)	% (95% CI)	% (95% CI)
LA County	8.4 (7.5, 9.3)	11.1 (9.6, 12.7)	5.7 (4.7, 6.7)
Race/ethnicity			
White	12.8 (10.4, 15.1)	14.6 (11.1, 18.2)	10.6 (7.8, 13.5)
Latino	6.1 (5.0, 7.3)	9.0 (7.0, 11.1)	3.4 (2.5, 4.4)
Black	5.8 (3.8, 7.9)	7.3 (3.8, 10.8)⁎	4.4 (2.3, 6.6)⁎
Asian	8.9 (6.3, 11.4)	11.1 (7.3, 15.0)	6.7 (3.4, 9.9)⁎
Education			
Less than high school	4.6 (2.8, 6.5)	7.3 (3.8, 10.8)⁎	2.4 (0.7, 4.1)⁎
High school	8.1 (6.2, 10.1)	12.3 (8.9, 15.7)	3.6 (2.1, 5.2)
Some college or higher	10.2 (8.8, 11.5)	12.3 (10.1, 14.4)	8.1 (6.5, 9.8)
Household income^FPL^			
0–99% FPL	6.3 (4.6, 7.9)	9.8 (6.3, 13.4)	3.9 (2.5, 5.3)
100–299% FPL	8.0 (6.5, 9.5)	10.9 (8.4, 13.3)	5.0 (3.6, 6.5)
≥300% FPL	10.7 (8.9, 12.5)	12.7 (10.1, 15.3)	8.0 (5.9, 10.2)

^FPL^Based on U.S. Census 2013 Federal Poverty Level (FPL) thresholds.

a Calculated Using 2000 U.S. census standard population. Seven age groups were used for age adjustment among adults: 18–24, 25–29, 30–39, 40–49, 50–59, 60–64, and 65 or over.

⁎ The estimate is statistically unstable (relative standard error > 30%) and therefore may not be appropriate to use for planning or policy purposes.

### 3.4 Factors associated with ever e-cigarette use

Table 3 presents results for unadjusted prevalence of ever e-cigarette use and results from our logistic regression models stratifying by gender.

**Table 3:** Unadjusted prevalence, logistic regression adjusted odds ratios (OR) of ever e-cigarette use among LA County adults, overall, and by gender.

Characteristic	Overall^LA^	Male^M^		Female^F^	
	% (95% CI)	% (95% CI)	Adjusted OR	% (95% CI)	Adjusted OR
LA County	8.6 (7.6, 9.5)	11.6 (9.9, 13.2)		5.7 (4.6, 6.7)	
Age (years)					
50+	3.3 (2.5, 4.0)	4.4 (3.1, 5.7)	1.00	2.3 (1.6, 3.0)	1.00
25–49	10.0 (8.4, 11.5)	13.1 (10.5, 15.7)	3.33 (2.40, 4.63)	6.8 (5.2, 8.4)	4.52 (2.92, 7.00)
18–24	18.5 (14.5, 22.5)	24.7 (18.5, 31.0)	11.98 (7.89, 18.21)	12.0 (7.4, 16.7)	15.91 (8.99, 28.17)
Gender					
Female	5.7 (4.6, 6.7)				
Male	11.6 (9.9, 13.2)				
Race/ethnicity					
White	10.1 (8.3, 11.8)	12.2 (9.4, 14.9)	1.00	7.9 (5.9, 9.9)	1.00
Latino	7.4 (6.1, 8.8)	11.3 (8.8, 13.9)	0.92 (0.68, 1.24)	4.0 (2.8, 5.2)	1.24 (0.81, 1.90)
Black	5.5 (3.6, 7.4)	7.4 (3.8, 10.9)	0.47 (0.28, 0.79)	4.0 (2.1, 5.9)	0.39 (0.20, 0.77)
Asian	10.4 (7.2, 13.6)	13.1 (8.3, 17.8)	1.35 (0.93, 1.95)	7.3 (3.2, 11.4)	2.35 (1.39, 3.97)
Nativity					
US born	10.8 (9.5, 12.2)	13.8 (11.5, 16.0)	1.00	8.0 (6.5, 9.4)	1.00
Foreign born	5.7 (4.4, 7.1)	8.7 (6.4, 11.1)	0.56 (0.42, 0.75)	3.0 (1.5, 4.4)	0.65 (0.44, 0.98)
Disability^DIS^					
No	8.1 (7.0, 9.2)	11.9 (9.9, 13.9)	1.00	4.5 (3.5, 5.6)	1.00
Yes	10.1 (8.1, 12.1)	10.6 (7.6, 13.5)	1.14 (0.85, 1.54)	9.7 (7.0, 12.4)	2.54 (1.77, 3.65)
Education					
Less than high school	4.2 (2.6, 5.8)	7.2 (3.9, 10.5)	1.00	1.8 (0.6, 3.0)⁎	1.00
High school	9.2 (7.1, 11.3)	13.9 (10.3, 17.5)	1.46 (0.98, 2.18)	4.0 (2.3, 5.7)	0.94 (0.47, 1.91)
Some college or higher	10.1 (8.7, 11.5)	12.2 (10.0, 14.5)	1.52 (1.02, 2.26)	8.0 (6.4, 9.7)	2.77 (1.50, 5.09)
Household income^FPL^					
0–99% FPL	7.2 (5.3, 9.1)	11.2 (7.3, 15.1)	1.00	4.6 (2.8, 6.3)	1.00
100–299% FPL	8.5 (6.9, 10.1)	11.7 (9.1, 14.4)	0.76 (0.54, 1.06)	5.4 (3.7, 7.0)	1.04 (0.64, 1.69)
≥300% FPL	9.4 (7.8, 11.0)	11.6 (9.0, 14.1)	1.05 (0.73, 1.51)	6.8 (5.0, 8.7)	1.46 (0.86, 2.48)
Housing instability in 5 years^HI^					
No	8.1 (7.1, 9.1)	11.2 (9.5, 12.9)	1.00	5.2 (4.2, 6.2)	1.00
Yes	16.6 (11.7, 21.6)	17.5 (10.4, 24.6)	1.17 (0.75, 1.81)	15.5 (8.8, 22.1)	2.22 (1.20, 4.10)
Marijuana (Cannabis) use in the past year					
No	6.3 (5.4, 7.2)	8.6 (7.0, 10.2)	1.00	4.3 (3.3, 5.2)	1.00
Yes	25.4 (21.0, 29.7)	26.9 (21.2, 32.6)	1.73 (1.31, 2.28)	22.3 (16.1, 28.4)	1.89 (1.25, 2.85)
Alcohol drinking in the past month^AL^					
Non-drinkers	4.7 (3.6, 5.7)	7.2 (5.1, 9.3)	1.00	2.9 (1.9, 3.9)	1.00
Non-heavy or non-binge drinkers	8.3 (6.7, 9.9)	10.3 (7.7, 12.8)	1.38 (1.03, 1.85)	6.1 (4.1, 8.1)	1.52 (1.03, 2.24)
Heavy or binge drinkers	19.9 (16.5, 23.3)	21.7 (16.9, 26.4)	1.80 (1.33, 2.42)	16.9 (12.3, 21.4)	2.65 (1.72, 4.07)
Cigarette smoking status^CIG^					
Non-smoker/non-regular smoker	3.5 (2.6, 4.3)	5.5 (3.8, 7.1)	1.00	2.0 (1.2, 2.8)	1.00
Former smoker	13.1 (10.5, 15.8)	14.2 (10.5, 18.0)	4.64 (3.39, 6.35)	11.5 (8.1, 15.0)	9.58 (6.21, 14.76)
Current smoker	28.0 (23.8, 32.1)	27.8 (22.5, 33.2)	9.40 (6.94, 12.75)	28.3 (22.0, 34.6)	20.64 (13.33, 31.98)

^LA^An interaction between conventional cigarette smoking status and gender was significantly associated with e-cigarette use (p < 0.05).

^M^Multivariable logistic regression model among males, Hosmer-Lemeshow Goodness-of-Fit Test: X^2^ = 14.64, p = 0.067.

^F^Multivariable logistic regression model among females, Hosmer-Lemeshow Goodness-of-Fit Test: X^2^ = 5.42, p = 0.711.

^DIS^Disability was defined as a positive response to any of the following questions: “Are you limited in any way in any activities because of a physical, mental, or emotional problem?”; “Do you now have any health problem that requires you to use special equipment, such as a cane, wheelchair, a special bed or special telephone?”; “Do you consider yourself a person with a disability?”

^FPL^Based on U.S. Census 2013 Federal Poverty Level (FPL) thresholds.

^HI^Reported being homeless or not having their own place to live or sleep in the past 5 years.

^AL^Binge drinking for females is drinking 4 or more drinks and males 5 or more drinks on one occasion at least one time in the past month. Heavy drinking is males consuming >60 drinks and females >30 drinks in the previous month.

^CIG^Current smoker is an adult who has smoked 100 cigarettes in his or her lifetime and who currently smokes cigarettes every day or somedays; Non-regular smoker is an adult who has not smoked 100 cigarettes in his or her lifetime and who currently smokes cigarettes every day or somedays; Former smoker is an adult who has smoked at least 100 cigarettes in his or her lifetime but who had quit smoking at the time of interview.

⁎ The estimate is statistically unstable (relative standard error > 30%) and therefore may not be appropriate to use for planning or policy purposes.

For males, compared to males ages 50+, ever e-cigarette use was significantly higher among 18–24 years old males (OR = 11.98; 95% CI = 7.89, 18.21) and 25–49 years old males (OR = 3.33; 95% CI = 2.40, 4.63). The odds of ever e-cigarette use were significantly lower among black males (OR = 0.47; 95% CI = 0.28, 0.79) compared with white males and among foreign born males (OR = 0.56; 95% CI = 0.42, 0.75) compared with the US born males. Ever e-cigarette use was significantly higher among those with some college or higher education level (OR = 1.52; 95% CI = 1.02, 2.26) compared with less than a high school degree. Ever e-cigarette use was significantly higher among those who use marijuana (OR = 1.73; 95% CI = 1.31, 2.28), are heavy or binge drinkers (OR = 1.80; 95% CI = 1.33, 2.42) or are non-heavy or non-binge drinkers (OR = 1.38, 95% CI = 1.03, 2.42), and currently smoke cigarettes (OR = 9.40; 95% CI = 6.94, 12.75) or are former smokers (OR = 4.64; 95% CI = 3.39, 6.35).

For females, compared to females ages 50+, ever e-cigarette use was significantly higher among 18–24 year old females (OR = 15.91; 95% CI = 8.99, 28.17) and 25–49 year old females (OR = 4.52; 95% CI = 2.92, 7.00); compared to white females, the odds of ever e-cigarette use was significantly lower among black females (OR = 0.39; 95% CI = 0.20, 0.77) and significantly higher among Asian females (OR = 2.35; 95% CI = 1.39, 3.97). Ever e-cigarette use was significantly higher among those ever homeless in the past 5 years (OR = 2.22, 95% CI = 1.20, 4.10). Additionally, compared to females without a disability, ever e-cigarette use was significantly higher among females with a disability (OR = 2.54; 95% CI = 1.77, 3.65); compared to less than high school graduate females, ever e-cigarette use was significantly higher among those with some college or higher (OR = 2.77; 95% CI = 1.50, 5.09); ever e-cigarette use was significantly higher among those who use marijuana (OR = 1.89; 95% CI = 1.25, 2.85), are heavy or binge drinkers (OR = 2.65; 95% CI = 1.72, 4.07) or are non-heavy or non-binge drinkers (OR = 1.52, 95% CI = 1.03, 2.24), and currently smoke cigarettes (OR = 20.64; 95% CI = 13.33, 31.98) or are former smokers (OR = 9.58; 95% CI = 6.21, 14.76).

## 4 Discussion

Our study findings highlight the relatively high levels (18.5%) of unadjusted ever e-cigarette use among young adults (ages 18–24 years) in Los Angeles County compared to what has been found in other studies in the US (14.8%) (Levy et al., 2017) and led to the identification of important correlates and gender-specific differences that can be used to guide intervention strategies (Choi and Forster, 2013; Sutfin et al., 2013). Overall, 8.6% of our Los Angeles County adult survey participants reported ever e-cigarette use. This overall prevalence is within the range of what has been reported in other U.S. national studies (Zhu et al., 2017), being higher than the prevalence reported in some studies (Levy et al., 2017; King et al., 2015), and lower than that reported in other studies (Wilson and Wang, 2017; Schoenborn and Gindi, 2015; Delnevo et al., 2016; Biener et al., 2015). In our study, more males reported ever e-cigarette use than females, while other published results are mixed, with most studies reporting a higher prevalence among males (Choi and Forster, 2013; Sutfin et al., 2013; Schoenborn and Gindi, 2015; Goniewicz and Zielinska-Danch, 2012), though two studies showed females with higher ever e-cigarette use than males (Levy et al., 2017; King et al., 2015).

Consistent with what has been widely reported, our study found that both males and females who were younger (<50 years old) (Wilson and Wang, 2017; Levy et al., 2017; King et al., 2015; Schoenborn and Gindi, 2015; Delnevo et al., 2016), had some college or higher education(Levy et al., 2017; King et al., 2015; Hartwell et al., 2017), used marijuana (Bluestein et al., 2019), engaged in alcohol drinking (Bluestein et al., 2019; Grant et al., 2019) and were former or current smokers (Wilson and Wang, 2017; Levy et al., 2017; King et al., 2015; Schoenborn and Gindi, 2015; Delnevo et al., 2016; Biener et al., 2015; Wang et al., 2016) were significantly more likely to have ever used e-cigarettes. Our study also found that those who were Black or foreign born were significantly less likely to have ever used e-cigarettes, which has rarely been reported elsewhere.

Household income was not associated ever e-cigarette use in our study, which is consistent with what has been reported in previous studies (Wilson and Wang, 2017; King et al., 2015). However, one study found a positive association between household income and ever e-cigarette use (Levy et al., 2017). The household income in that study was not categorized in the same way as in our study, so it is difficult to determine whether differences in the household income categorization may have contributed to the different findings.

Among females, those with a disability were more likely to report ever e-cigarette use, which was consistent with findings from other studies (Spears et al., 2016). Those who had experienced housing instability in the past 5 years were also more likely to report ever e-cigarette use. Finally our study found higher ever e-cigarette use among Asian females, which was not reported by other studies focusing on both genders and which concluded either no significant difference (King et al., 2015; Biener et al., 2015), or higher ever e-cigarette use in other ethnic groups such as Whites (Levy et al., 2017; Delnevo et al., 2016). It is possible that this reflects social or cultural differences in attitudes toward e-cigarette use specific to Asians in Los Angeles County compared to nationally. Los Angeles County has the largest county population of Asians in the US (Du et al., 2017), and further research is warranted to examine these differences in order to develop and implement culturally relevant interventions.

There are several limitations in the present study that deserve mention. First, the data are cross-sectional and cannot be used to infer causation. Second, the question by which e-cigarette use is measured is not the same question used in other major surveys (i.e. “Have you ever used an electronic cigarette or e-cigarette, even once or twice?”). By not including “even once or twice,” in the question wording, it is possible that the prevalence of ever e-cigarette use was underreported. Third, the data are self-reported, therefore, responses might be subject to recall bias or response bias due to social desirability. Fourth, while 22.4% of LA County Health Survey interviews were with cell phone only households, this representation is lower than the estimated 34.9% of cell phone only households found in LA County in 2015 (Abt and SRBI, 2017). This could have resulted in a skewed sample, and coverage bias could result from not including sufficient representation of households with cell phones only. However, statistical weighting was used to generalize the sample survey data to the overall LA County population. Fifth, the low response rate may have introduced bias if the non-responders had different sociodemographic characteristics and behaviors than did the responders. However, studies have demonstrated that non-response does not necessarily introduce substantial bias into survey estimates (Keeter et al., 2000; S et al., 2006). Finally, the variables of past 30-day or current e-cigarette use were not included in our study due to small sample sizes.

## 5 Conclusion

These results underscore the emerging popularity of ever e-cigarette use among adults, particularly young adults. In LA County, age-adjusted prevalence of ever e-cigarette use was 11.1% among adult males and was 5.7% among adult females. Our analysis demonstrated that various sociodemographic factors as well as concurrent use of other substances are associated with ever e-cigarette use among Los Angeles County adults. These factors should be taken into consideration when developing evidence-based interventions and prevention programs targeting at-risk groups.

## Declaration of Competing Interest

All authors declare that they have no conflicts of interest.
